# [Expression of Concern] Halofuginone induces the apoptosis of breast cancer cells and inhibits migration via downregulation of matrix metalloproteinase-9

**DOI:** 10.3892/ijo.2025.5812

**Published:** 2025-10-13

**Authors:** Mei Ling Jin, Sun Young Park, Young Hun Kim, Geuntae Park, Sang Joon Lee

Int J Oncol 44: 309-318, 2014; DOI: 10.3892/ijo.2013.2157

Following the publication of the above paper, it was drawn to the Editor's attention by a concerned reader that, for the cell invasion assay experiments shown in Fig. 4B, the 'Con' and 'LPA+HF' data panels contained an overlapping section, such that data which were intended to show the results of differently performed experiments appeared to have been derived from the same original source.

The authors were contacted by the Editorial Office to offer an explanation for this possible anomaly in the presentation of the data in this paper, although up to this time, no response from them has been forthcoming. Owing to the fact that the Editorial Office has been made aware of potential issues surrounding the scientific integrity of this paper, we are issuing an Expression of Concern to notify readers of this potential problem while the Editorial Office continues to investigate this matter further.

